# Successful use of tablet personal computers and wireless technologies for the 2011 Nepal Demographic and Health Survey

**DOI:** 10.9745/GHSP-D-12-00056

**Published:** 2013-07-11

**Authors:** Deepak Paudel, Marie Ahmed, Anjushree Pradhan, Rajendra Lal Dangol

**Affiliations:** a U.S. Agency for International Development/Nepal, Kathmandu, Nepal; b U.S. Agency for International Development/Rwanda, Kigali, Rwanda; c ICF International, Calverton, MD, USA; d New ERA, Kathmandu, Nepal

## Abstract

Computer-Assisted Personal Interviewing (CAPI), coupled with the use of mobile and wireless technology, is growing as a data collection methodology. Nepal, a geographically diverse and resource-scarce country, implemented the 2011 Nepal Demographic and Health Survey, a nationwide survey of major health indicators, using tablet personal computers (tablet PCs) and wireless technology for the first time in the country. This paper synthesizes responses on the benefits and challenges of using new technology in such a challenging environment from the 89 interviewers who administered the survey. Overall, feedback from the interviewers indicate that the use of tablet PCs and wireless technology to administer the survey demonstrated potential to improve data quality and reduce data collection time—benefits that outweigh manageable challenges, such as storage and transport of the tablet PCs during fieldwork, limited options for confidential interview space due to screen readability issues under direct sunlight, and inconsistent electricity supply at times. The introduction of this technology holds great promise for improving data availability and quality, even in a context with limited infrastructure and extremely difficult terrain.

## Abstract (precis)

Using tablet personal computers and wireless technologies in place of paper-based questionnaires to administer the Nepal DHS in a geographically diverse setting appeared to improve data quality and reduce data collection time. Challenges include inconsistent electricity supply, safe storage and transport of equipment, and screen readability issues under direct sunlight, which limited confidential interview spaces.

## BACKGROUND

The advent of mobile and wireless technology has created countless opportunities to connect ideas and information that would never have been tapped with traditional technology, such as desktop computers or non-electronic mechanisms. As mobile and wireless technology has become more affordable, reliable, powerful, and user-friendly,^1^ its adoption has exploded, particularly in less-developed countries where access to infrastructure for information and communication technologies, such as landlines, is limited. Use of appropriate technology can play an important—and even transformative—role in providing more accurate and rapid information.^2^-^3^ This paper highlights lessons learned, benefits, and challenges of using Computer-Assisted Personal Interviewing (CAPI) and wireless technology to collect data for a large-scale survey in Nepal—a geographically difficult and resource-poor environment.

Ranked 157^th^ of 187 countries in the Human Development Index,^4^ Nepal features a small territory (147,181 sq km) with diverse geography and a relatively small population (26.6 million)^5^ but with ethnic, religious, and cultural diversity that adds another layer of development challenges.

The country has also been wracked by a decade-long conflict that nominally ended in 2006, but the country remains fragile politically. Communications and transportation systems are improving but are still poor. Only 43% of the population has access to all-weather roads.^6^ The majority of the population (67%) has access to some electricity, but it may not be available 24 hours per day year-round.^5^ Nepal currently has about 10 million telecommunications users, 89% of whom use mobile phones.^7^ In contrast, in 2000, there were only 300,000 telecommunications users, with less than 4% using mobile phones.^8^

The Demographic and Health Survey (DHS) is a nationally representative survey that provides reliable data on population and family planning, health, HIV, and nutrition in more than 90 countries, using mostly paper questionnaires.^9^

Nepal conducted the DHS in 1996, 2001, and 2006 using paper questionnaires. Nationwide data collection with paper-based questionnaires usually takes 5 to 6 months, and data entry, which overlaps with the fieldwork, is completed a month after completing the fieldwork. The final report is available approximately one year after concluding the fieldwork.

In 2011, ICF International (an international organization that provides technical assistance to carry out the DHS) and New ERA (a local research firm in Nepal that specializes in large-scale surveys), in collaboration with the Ministry of Health and Population, conducted the DHS in Nepal using tablet personal computers (tablet PCs); the preliminary report was published within 2 months, and the final report within 9 months, after data collection. The objective of transitioning from paper questionnaires to tablet PCs was to ensure higher-quality data collection and timely reporting.

Use of microcomputers closer to the point of data collection has proved to significantly reduce error rates and produce data quickly.^1^ Nepal is the second country in the world to use tablet PCs to collect DHS data and the first country to develop and use a non-Latin script for the DHS using tablet PCs.

Using microcomputers for data collection reduces error rates and collection time.

## THE TOOL AND TECHNOLOGY

The Nepal DHS (NDHS) team initially considered using a number of different tools for the 2011 data collection effort, including personal digital assistants (PDAs), iPhones, iPads, tablet PCs, Android tablets, and standard laptops. We eliminated PDAs and iPhones, with their small screen sizes, as options because of the length and complexity of the DHS questionnaire. We also eliminated Android tablets and iPads because they were incompatible with the Windows-based DHS software, CSPro (Census and Survey Processing System). Compared with tablet PCs, standard laptops are heavier, susceptible to humidity and dust, and have a relatively short battery life, so the tablet PC emerged as the best option. We selected the ASUS Tablet PC T101MT based on cost, local availability, battery life, and user-friendliness. Specifications of ASUS PC T101MT include: Intel ATOM N450 CPU processor, 10.1-inch touchscreen display (1024 x 600) with PenWrite technology; 1.3 kg weight, dimensions of 26.4 × 18.1 × 3.1 cm, and average battery life of 6.5 hours.

A tablet PC is a portable personal computer equipped with a touch screen as a primary input device. Generally, tablet PCs have an integrated wireless adapter for connecting to the Internet and local networks and Bluetooth for transferring data over short distances.

Generally, the interviewer used a stylus to enter the response and the optional keyboard when the stylus was not working properly. However, supervisors used the keyboard the majority of the time while reviewing the data.

Nepal's mobile network uses both Global System for Mobile Communications (GSM) and Code Division Multiple Access (CDMA) technology. We chose to use the CDMA2000 1X network, provided by Nepal Telecom, based on its wider coverage, especially in rural areas (2,765 of 3,915 village development committees have CDMA coverage). Using the CDMA network, field workers transferred data from the field to the main office via the Internet using a protocol developed by ICF International called Internet File Streaming System (IFSS). We also used IFSS to deploy software updates from the central server to supervisors and interviewers in the field.

## THE TEAM

The survey organization team trained and mobilized 65 interviewers, 16 supervisors, and 8 data quality assurance supervisors, divided into 16 teams. Selection of interviewers and supervisors consisted of initial screening, a written examination, practical exercises on using computers, and personal interviews.

Candidates had to be computer literate, which we defined as having the ability to operate basic computer functions without any specific guidance and having some experience using computers for other purposes. We invited 89 candidates (10 more than what was needed to account for potential dropouts) to participate in the training program. As a result of the computer literacy requirement, the demographic profile of the selected fieldworkers was different than that of the fieldworkers from previous NDHSs using paper questionnaires. Approximately 35% of the field staff for the 2011 DHS was under 25 years of age, and over half were under the age of 30. Interviewers possessed at least a bachelor's degree in fields such as population studies, sociology, and public health. Only 15% of the 89 interviewers and supervisors for the 2011 NDHS had participated in data collection for a previous NDHS.

After extensive training, including intensive coaching for some interviewers on how to use tablet PCs and how to navigate different window screens, we deployed the field workers in 16 teams in Kathmandu Valley, where the research team could monitor and mentor all the field workers closely during the early stage.

## THE PROCESS

Using CAPI, the interviewer enters the respondents' responses directly into a computer database. CAPI provides several advantages, such as:

- ability for the interviewer to select the appropriate language in which to administer the questionnaire
- automatic skip-pattern and validation of the response, minimizing data-entry errors
- drop-down menus to select appropriate options

The interviewers entered data directly into the tablet PC during each interview and submitted the data to their respective supervisor at the end of each day using the Bluetooth file transfer system. The supervisors reviewed the data for inconsistencies and provided immediate feedback to the interviewers, for example, to check whether the correct identity and line number were entered so that data could be linked properly or to identify other inconsistencies that could not be captured with the computer program, such as matching the respondent's name and sex.

Once data quality was assured, the supervisor transferred the data to the central office using IFSS through the CDMA network. The database manager in Kathmandu then checked the data reported by the field teams and provided feedback as soon as possible via mobile phones if inconsistencies were detected (see Figure).

**Figure:**
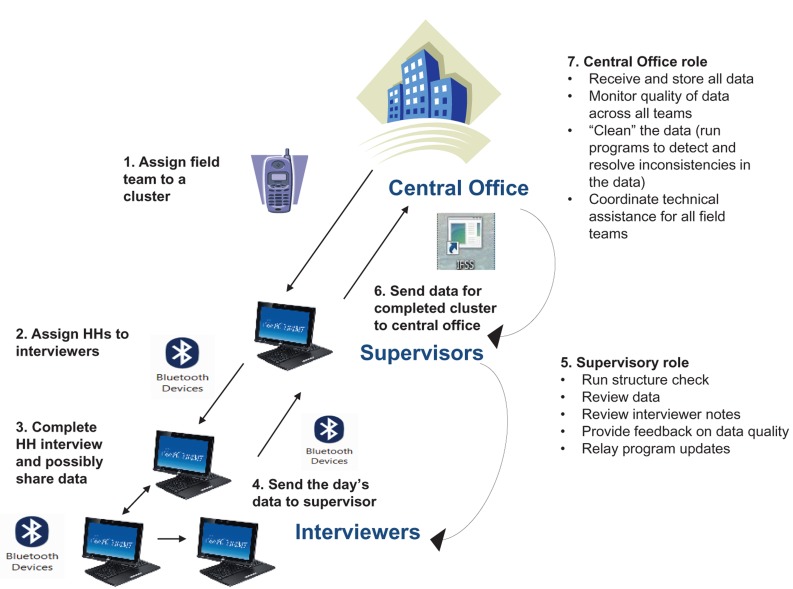
Data Collection and Reporting Scenario Abbreviations: HH, household; IFSS, Internet File Streaming System. In the 2011 Nepal DHS, interviewers collected household data on their tablet PCs and submitted it to their supervisors using Bluetooth technology. Supervisors provided immediate feedback to the interviewers, when necessary, and transferred the data to the central office using a protocol called Internet File Streaming System. Illustration prepared by ICF International.

Data collection for the NDHS started in February 2011. After completing 1 month of data collection, we organized review sessions to discuss progress, challenges, benefits, and areas for improvement related to data collection and use of the tablet PCs, Bluetooth, and IFSS system. Since all interviewers were trained on how to use the paper surveys as a backup to the tablet PC, they were able to compare the two experiences even if they had not participated in the previous paper-based DHS.

We also asked interviewers about respondents' perceptions of the tablets, logistical challenges, connectivity and data transfer challenges, and other technical issues. During the review sessions, we provided any necessary technical support and guidance to the field teams on the use of tablet PCs and wireless technology, the interviewing process, and other logistical issues.

## BENEFITS TO USING CAPI

We identified several benefits—some unanticipated—to using tablet PCs during our discussions with the field workers.

### Improved Data Quality

The most important benefit to using tablet PCs for data collection, as perceived by 80% of the interviewers, was that the survey software guided the interview, improving the interview experience, primarily because it was easier to administer the questionnaire and ensure data quality. For example, the built-in skip patterns in the electronic questionnaire reduced the chance of missing questions or following incorrect skip patterns. Also, the automatic error messages alerted interviewers to any inconsistencies in the information provided by the respondents, such as birth of a child before the date of first sex. The interviewers could resolve these inconsistencies immediately, reducing the chances of collecting inconsistent data.

Built-in skip patterns and automatic error messages in the electronic questionnaire improved data quality.

### Reduced Data Collection Time

The majority of interviewers (70%) indicated anecdotally that the electronic format saved time in conducting the interview because of the built-in skip patterns, filters, and auto-fill features (with calendars, for example). Implementing CAPI reduced the overall time needed for data collection by approximately one month (6.5 months for data collection in 2006 compared with 5.5 months in 2011). Elimination of data-entry redundancy with paper questionnaires contributed to the time savings. In addition, new data could be aggregated and checked for consistency on a daily basis.

### Ease of Handling

Field interview teams quickly learned how to handle the tablet PCs without difficulty, with 68% of interviewers indicating that use of the tablets was straightforward and that they could move through the questionnaires easily. The remaining interviewers were able to manage the tablets after some practice.

Overall, use of Bluetooth and IFSS was also smooth. The connection to the CDMA network took, on average, one minute and data transfer to the server in Kathmandu took approximately 5 to 7 minutes. In contrast, in the past, paper-based surveys had to be sent to Kathmandu via pouch mail or hand-carried, which took days or even weeks.

Interviewers also cited the convenience of carrying the tablet PCs instead of cumbersome paper questionnaires, an expected benefit particularly in the remote hilly and mountainous areas where interviewers may have to hike for several hours or days to reach respondents.

Tablet PCs can also be used in low-light situations, so interviewers were able to work during evening hours, which would have been a challenge with paper questionnaires due to frequent power outages.

Use of tablet PCs can also be helpful to switch languages during interviews, compared with paper questionnaires.

### Improved Feedback Loop

Using tablet PCs for data collection and daily data transfer offered a stronger feedback loop between interviewers and the central backstop team than with paper questionnaires. The NDHS backstop team was able to provide relevant feedback to the interviewers faster, which improved data quality, resulting in less frequent missing and inconsistent values. Prompt feedback also motivated interviewers to track their own progress toward targets more effectively.

Using tablet PCs and wireless technology facilitated prompt feedback between fieldworkers and the central backstop team.

### Positive Perception of Respondents

Interviewers reported that survey respondents were curious about being interviewed using the tablet PCs. The interviewers perceived a high level of respect and enthusiasm from respondents, and they felt that respondents viewed them as technical employees with higher education. This was an unanticipated, but encouraging, finding, especially because of respondents' limited exposure to computers.

Interviewers also reported that respondents perceived that the use of technology validated the DHS data collection process, and respondents were more willing to participate in the survey due to the perceived importance of the survey. Some respondents told interviewers that they felt the electronic process was more confidential than paper-based forms because the electronic versions could not be readily seen by others.

**Figure:**
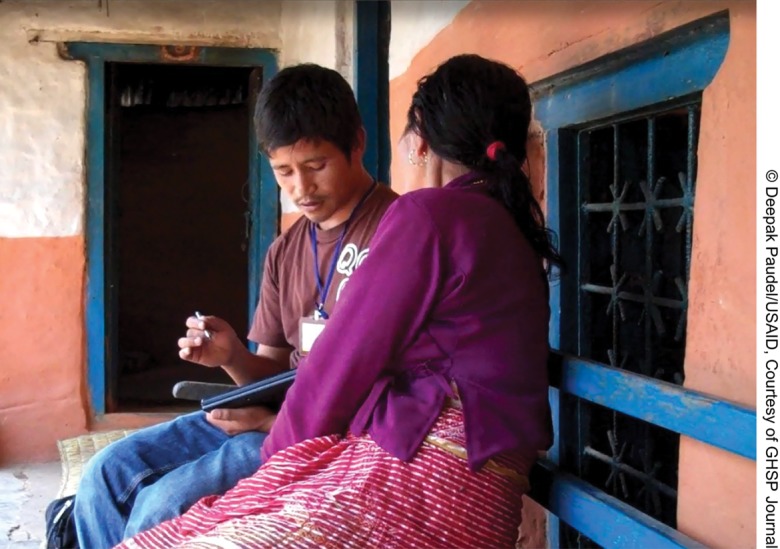
An interviewer uses his tablet PC to collect responses from a rural woman.

## CHALLENGES WITH USING CAPI

Although the use of tablet PCs has been encouraging, the innovation has not been without its challenges.

### Storage and Transport

A key challenge was the storage and transport of tablet PCs during fieldwork. The interviewers conducted their fieldwork in community clusters, where news of the tablet PCs can spread quickly, adding a dimension of security concerns and requiring interviewers to be vigilant because they were accountable as a team for any theft or damage. Safe storage was a particular challenge in remote areas because some interviewers had to stay in community members' homes.

Safe storage and transport of the tablet PCs during fieldwork was a key challenge.

To alleviate these challenges, the interviewers traveled in teams and so they could support each another in protecting the equipment. In addition, team members were trained to lock and be aware of their tablet PCs at all times, even during meal and rest times.

Interviewers also expressed concerns about protecting the equipment from rain, particularly of concern during the monsoon season. However, we concluded fieldwork by June 2011, just prior to the monsoon season.

### Limited Confidential Interview Environment

Because tablet PC screens are difficult to read outdoors, particularly under direct sunlight, it limited the options for confidential interview space. Interviewers often had to conduct interviews inside the home, where it was a challenge to maintain privacy due to small living spaces and thin walls in rural Nepali homes. This was of particular concern when asking sensitive questions related to sexual behavior and domestic violence. The tablet PCs also sometimes attracted other people who were curious about the technology. The interviewers often had to make extra effort to maintain privacy, which usually demanded more time to administer the questionnaire.

### Consistent Availability of Electricity

Nepal relies primarily on hydropower, and so electricity shortages increase significantly in most areas of the country during the dry season between February and April—the time period during which we collected data. Some areas were without electricity for more than 14 hours per day. Recharging the tablet PCs in this environment was a major impediment.

We equipped interviewers with extra batteries that had approximately 6.5 hours of charge per battery, which temporarily addressed the issue of low battery power during interviews. But teams still had to find a power source to charge all the batteries and their mobile phones at the end of the day.

Solar chargers were not practical because interviewers were busy using their tablet PCs during the sunlight period. Therefore, as a contingency plan, interviewers carried a few paper questionnaires and extra batteries to use as needed. We also provided portable generators to interviewer teams located in areas lacking electricity.

Backup plans in case of power outages must be put in place in resource-constrained environments.

### Other Challenges

Although acceptability of the tablet PCs was high among respondents, there were a few cases of skepticism. As part of the informed consent process, respondents were informed that the interview would not be video- or audio-recorded and that the recording feature had been disabled on the tablet PCs. However, a few respondents were still concerned.

We also had to make minor revisions in the data-entry software once the team was in the field, for example, to increase the maximum number of family members that the interviewer could enter into the database. We made these changes at the central office in Kathmandu and provided the updated files to the interviewers through IFSS.

**Figure:**
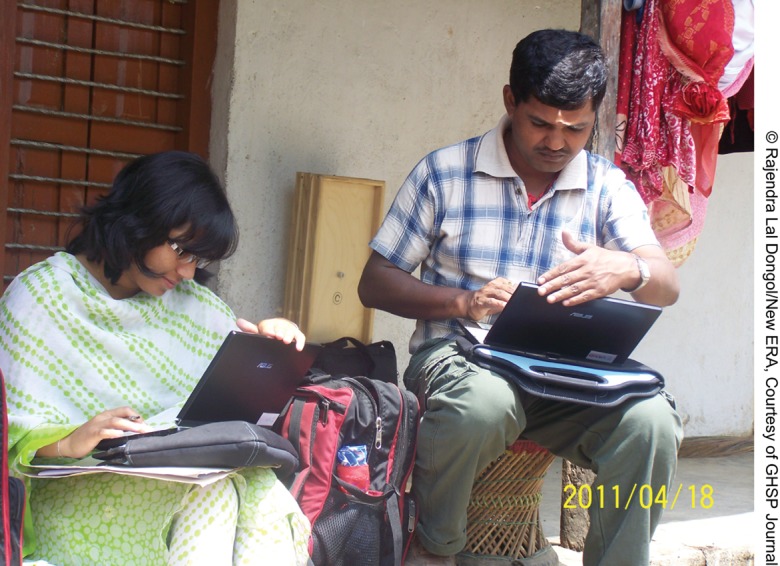
Interviewers transfer data between tablet PCs.

## LESSONS LEARNED

Key lessons learned from our experience in Nepal:

**Selection and training of interviewers:** Proper selection and thorough training of interviewers is crucial for the success of the program. The interviewers received 5 weeks of training, 1 week longer than previous DHS training for paper questionnaires. The training covered interviewing skills, types of questions in the DHS, administration of the paper questionnaire, and proper use and handling of tablet PCs, Bluetooth, and IFSS. Examples of issues that may affect the quality of the interview experience and data collection, but that can be resolved through training, include the pace of checking completed forms, awareness of battery life, and calibrating the touch screen to ensure the stylus marks the correct responses.

**Equipment care:** Training on proper handling and care of the equipment is also very important, particularly in a rural context where the equipment has to be transported through rough terrain, the power supply is not stable, and unexpected rain is a concern. We provided teams with generators, rain shields, umbrellas, and several other items to manage these challenges. Enforcing joint responsibility for theft of, or damage to, the tablet PCs among the interviewer teams helped to ensure security of the tablets during transport and storage. With proper care and maintenance, these tablet PCs (and portable generators) can be reused in future surveys, resulting in additional cost savings over the long term.

**Maintaining data security:** Data security is a prime issue; daily backup of the data should be done properly. After completing each interview, the data were automatically saved to an external data card on each tablet PC. We also provided team supervisors with flash drives for daily data backup, in addition to daily data transmission to the central database server.

**Immediate feedback on data quality:** Regular, often immediate, feedback helped to resolve technology and survey-related problems in a timely fashion, resulting in improved data quality. Review meetings that focused on both individual and team issues provided additional opportunities for feedback and practical problem solving.

**Adequate preparation:** Sufficient time must be allocated to designing and pretesting the electronic questionnaire and to overall testing and debugging the software, particularly for questionnaires in multiple languages and in a non-Latin script, as was the case in Nepal. DHS questionnaires are lengthy and complex, so it is crucial to ensure that the question flow and skip patterns function correctly before using them in the field.

**Local purchase:** Purchasing the tablet PCs and accessories locally facilitated more efficient servicing of equipment than if we had purchased the equipment internationally. Although new technology, such as tablet PCs, may be more expensive in less-developed countries such as Nepal, the probable cost saved in shipping and delays can more than make up for that difference.

## CONCLUSION

The use of tablet PCs and wireless technology to administer a complex survey in Nepal has demonstrated potential to improve data quality and reduce data collection time. These benefits outweigh manageable challenges, such as inconsistent electricity supply, storage and transport of the tablet PCs, and limited confidential interview environment. Use of technology holds great promise for improving data availability and quality, even in a context with limited infrastructure and difficult terrain. Because use and evolution of such technology is growing rapidly, it may be helpful to carry out further detailed research, including cost-benefit analysis, to precisely report the time, cost, and human resource savings and improved data quality through the use of technology.[Fig f04]

**Figure f04:**
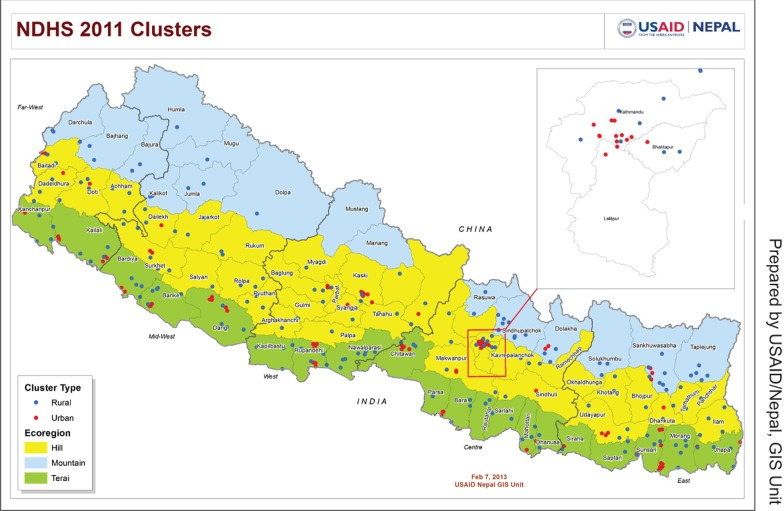
The 2011 Nepal Demographic and Health Survey (NDHS) is a nationally representative survey covering both rural and urban areas.
